# Osteogenic Matrix Cell Sheet Transplantation Enhances Early Tendon Graft to Bone Tunnel Healing in Rabbits

**DOI:** 10.1155/2013/842192

**Published:** 2013-09-11

**Authors:** Yusuke Inagaki, Kota Uematsu, Manabu Akahane, Yusuke Morita, Munehiro Ogawa, Tomoyuki Ueha, Takamasa Shimizu, Tomohiko Kura, Kenji Kawate, Yasuhito Tanaka

**Affiliations:** ^1^Department of Orthopaedic Surgery, Nara Medical University, Shijocho 840, Kashihara, Nara 634-8522, Japan; ^2^Department of Public Health, Health Management and Policy, Nara Medical University, Shijocho 840, Kashihara, Nara 634-8522, Japan; ^3^Department of Biomedical Engineering, Doshisha University, Tataramiyakodani 1-3, Kyotanabe, Kyoto 610-0394, Japan; ^4^Department of Artificial Joint and Regenerative Medicine for Bone and Cartilage, Nara Medical University, Shijocho 840, Kashihara, Nara 634-8522, Japan

## Abstract

The objective of this study was to determine whether osteogenic matrix cell sheets (OMCS) could induce bone formation around grafted tendons, thereby enhancing early stage tendon to bone tunnel healing in skeletally mature male Japanese white rabbits. First, the osteogenic potential of rabbit OMCS was evaluated. Then, the OMCS were transplanted into the interface between the grafted tendon and the bone tunnel created at the tibia. Histological assessments and biomechanical tensile testing were performed after 3 weeks. The rabbit OMCS showed high alkaline phosphatase (ALP) activity, positive staining of ALP, and osteogenic potential when transplanted subcutaneously with beta tricalcium phosphate disks. Newly formed bony walls and positive collagen type I staining were seen around the grafted tendon with OMCS transplantation, whereas such bony walls were thinner or less frequent without OMCS transplantation. Micro-computed tomography images showed significantly higher bone volume in the OMCS transplantation group. The pullout strength was significantly higher with OMCS (0.74 ± 0.23 N/mm^2^) than without OMCS (0.58 ± 0.15 N/mm^2^). These results show that OMCS enhance early tendon to bone tunnel healing. This method can be applied to cases requiring early tendon to bone tunnel healing after ligament reconstruction surgery.

## 1. Introduction

Anterior cruciate ligament (ACL) reconstruction using a hamstring tendon graft has been a common procedure in sports medicine [1]. Osteoblast formation around the grafted tendon plays an important role in the healing process at 1 to 2 weeks after the tendon graft surgery [2]. Rodeo et al. [3] described collagen fiber continuity to the bone lining of the newly formed bone as “Sharpey's fibers.” A relatively long time is necessary for the establishment of collagen fiber continuity. Consequently, aggressive rehabilitation after surgery is avoided until the grafted tendon has matured and the interface between the grafted tendon and bone tunnel has been integrated [4, 5]. Although alternative treatment options to speed up the healing between tendon and bone have been studied, for example, using periosteum, calcium phosphate, hyperbaric oxygen and growth factors, or mesenchymal stem cells (MSCs) [6–12], there is a need for practical treatment options that enhance healing.

Recently, MSCs have been widely investigated with a variety of scaffolds as a cell source for tissue regeneration. In the field of ligament reconstruction, Lim et al. [12] reported that coating tendon grafts with MSCs in fibrin glue enhanced tendon graft integration. However, there are limitations to the use of MSCs with scaffolds, including possible immunological responses and biocompatibility [13]. Therefore, a scaffold-free technique for MSC transplantation may be ideal.

We previously developed a mechanical retrieval method for preparing cell sheets from rat MSCs, designated as osteogenic matrix cell sheets (OMCS). OMCS can be transplanted scaffold-free, resulting in in vivo bone formation in a rat model [14]. In the present study, we expanded on these results using a rabbit model. First, we assessed the osteogenic potential of OMCS from rabbit bone marrow MSCs both in vitro and in vivo and then evaluated whether OMCS enhance the grafted tendon to bone tunnel healing in order to facilitate ligament reconstruction surgeries, such as ACL reconstruction.

## 2. Materials and Methods

### 2.1. Animals

 A total of 16 skeletally mature male Japanese white rabbits, weighing 3.1 ± 0.2 kg, were purchased from Japan SLC (Shizuoka, Japan). Three rabbits were used for in vitro and in vivo assessment of OMCS, described below. The remaining 13 rabbits underwent tendon transplantation surgery, after which three rabbits were used for histology and 10 rabbits for biomechanical evaluation. The animal experimental protocol was approved by the Animal Care and Use Committee of the author's institute.

### 2.2. Preparation of Osteogenic Matrix Cell Sheets

**Figure 1:**
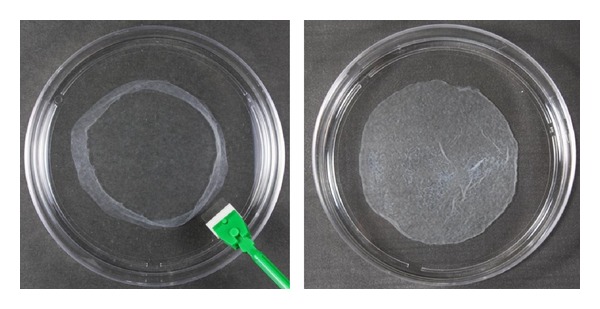
Photographs of rabbit OMCS. Sheets could be easily detached from the culture dish using a scraper (left). A sheet fully detached from the 10 cm culture dish (right).

The detailed method of OMCS preparation was previously reported [14–17]. Briefly, primary bone marrow cells aspirated from the humeral heads of rabbits were cultured in regular medium consisting of Earle's Minimal Essential Medium (Nacalai Tesque, Kyoto, Japan) with 15% fetal bovine serum (JRH Bioscience, Lenexa, KS) and antibiotics (100 U/mL penicillin and 100 *μ*g/mL streptomycin; Nacalai Tesque) for 2 weeks [18, 19]. After reaching confluence, the cells were released and seeded at 1 × 10^4^ cells/cm^2^ onto 10 cm culture dishes (Falcon, BD Biosciences, Franklin Lakes, NJ) in the above medium supplemented with 10 nM dexamethasone (Dex; Sigma, St. Louis, MO) and 82 *μ*g/mL ascorbic acid phosphate (Asap; L-ascorbic acid phosphate magnesium salt *n*-hydrate; Wako Pure Chemical Industrials, Kyoto, Japan) for 14 days. Although *β*-glycerophosphate (*β*-GP) was added to create OMCS in our initial study [14], recent investigations have revealed that OMCS with high osteogenic potential can be prepared without *β*-GP [16, 17]. Therefore, the OMCS in the present study were created without *β*-GP. The OMCS were easily detached from the culture dish by mechanical retrieval with a scraper and lifted with a pair of tweezers (Figure 1).

### 2.3. Alkaline Phosphatase Activity

The OMCS (the OMCS group) and cells cultured with regular medium (the control group) were used for measurement of alkaline phosphatase (ALP) activity, as reported previously [20]. Briefly, the OMCS and control cells were scraped into Nonidet P-40 (Nacalai Tesque) containing 1 mM MgCl_2,_ homogenized and centrifuged at 13,000 rpm (10,000 ×g) for 10 min at 4°C. The supernatant was assayed for ALP activity using *p*-nitrophenylphosphate (Nacalai Tesque) as a substrate. A 10-*μ*L aliquot of the supernatant was added to 1 mL of 50 mM *p*-nitrophenylphosphate containing 1 mM MgCl_2_ and the mixture was incubated for 30 min at 37°C. Next, 2 mL of 0.2 N NaOH was added to stop the enzymatic reaction and the absorbance at 410 nm was measured with a spectrophotometer. ALP activity was represented as the concentration of *p*-nitrophenol (*μ*M) released after 30 min of incubation at 37°C. Four wells of a 12-well plate (Falcon) were used for each group (quadruplicate), and this experiment was repeated three times with cells from three different animals.

### 2.4. Alkaline Phosphatase Staining

 OMCS and control cells cultured for 14 days in 6-well plates were used for ALP staining [20]. Briefly, the cells were rinsed twice with phosphate-buffered saline (PBS; Gibco, Paisley, UK) and stained with naphthol-AS-MX phosphate sodium salt (Sigma) and fast red violet LB salt (Nacalai Tesque) at room temperature for 10 min. Each group was analyzed in two wells (duplicate) and the experiment repeated three times with cells from three different animals.

### 2.5. In Vivo Assessment for Osteogenic Matrix Cell Sheet

 To evaluate the osteogenic potential in vivo, the OMCS were transplanted with beta tricalcium phosphate (*β*-TCP) disks (Hoya, Tokyo, Japan: 75% porosity, 5 mm diameter and 2 mm thickness). Briefly, the *β*-TCP disks were wrapped with the OMCS and then autologously implanted atsubcutaneous site on the back of the rabbits [15, 21]. The *β*-TCP disks alone were also implanted as a control. The disks were harvested after 4 weeks and decalcified in K-CX solution (Falma Co., Tokyo, Japan), embedded in paraffin, cut at the middle of the specimen, and stained with hematoxylin and eosin (H&E). Four high power field (×400) microscopic images were taken by digital camera from one section. The amount of new bone was quantified as a percentage of the bone area to the total area within the images using image analysis software, Image J (National Institute of Health, MD, USA) [22]. We repeated the experiment using bone marrow MSCs from three rabbits. Two samples were made from a rabbit; totally each group included six samples from three different rabbits.

### 2.6. Tendon Transplantation into the Bone Tunnel with the Osteogenic Matrix Cell Sheets

We modified the extra-articular tendon-bone healing model reported by Chen et al. [6, 11] to assess whether OMCS enhance the grafted tendon to bone tunnel healing. Briefly, knee joints were accessed via a lateral parapatellar approach. The long digital extensor tendon (EDL) was identified and cut to detach it from the lateral femoral condyle. A 3.0 mm diameter tunnel was created in the proximal tibial metaphysis. The proximal end of the EDL was passed through the tunnel and sutured at the medial aspect of the proximal tibia. For the right knees, the EDL was enveloped with an autologous OMCS and transplanted into the bone tunnel (the OMCS group). The same operation without the OMCS was performed on the left knees as a control group. Before passing the tendon into the bone tunnel, OMCS were sutured to the grafted tendon by nylon fiber at the proximal end of the tendon. After passing the tendon, we confirmed the presence of OMCS at the inlets and outlets of the bone tunnel. Animals were not immobilized and allowed free activity in their cages. All operations were performed by the same knee surgeon (Yusuke Inagaki).

### 2.7. Histology and Immunohistochemistry of Tendon Graft to Bone Tunnel Healing

 The proximal tibiae were harvested 3 weeks after the operation [11, 19]. The tibiae for histological evaluation were fixed in 10% neutral buffered formalin, decalcified in EDTA solution, and embedded in paraffin. Sections of 5 *μ*m thickness were made in planes parallel to the long axis of the tendon and stained with HE.

Commercially available mouse monoclonal antibody against human collagen type I (1 : 1000 dilution, Daiichi Fine Chemical, Takaoka, Japan) was used for immunohistochemistry [23, 24]. The secondary antibody was peroxidase-labeled goat antibody against murine immunoglobulin (Nichirei Bioscience, Tokyo, Japan). The manufacturer's information indicated that the antibody had cross-reactivity with rabbit collagen. The sections were dewaxed and immersed in methanol with 0.3% H_2_O_2_ to block endogenous enzyme activity. After blocking nonspecific reactions, the sections were incubated with primary antibody at 4°C overnight and then with secondary antibody at room temperature for 10 min. The color was developed using diaminobenzidine solution and the nuclei were lightly stained with hematoxylin.

### 2.8. Image Analysis and Biomechanical Tensile Test

At 3 weeks after the operation [11, 19], 10 rabbits were sacrificed for biomechanical tensile tests. After harvesting proximal tibiae, all soft tissues except the EDL were removed. Specimens were then wrapped in saline-soaked gauze and stored at −20°C until evaluation. Before evaluation, all specimens were thawed overnight at 4°C. Bone tunnel length was measured using a micro-computed tomography system (micro-CT, SMX-160CTS, Shimadzu, Kyoto, Japan). Furthermore, using micro-CT imaging software TRI/3D-BON (Ratoc System Engineering, Tokyo, Japan), slices of the perpendicular planes of the long axes of the bone tunnels at the midpoints were reconstructed. Next, areas of new bone formation around the grafted tendons and the major/minor axes of the cross section of the grafted tendon were measured using Image J. Finally, the cross-sectional area and the perimeter of the tendon were calculated by fitting to an ellipse [25].

After micro-CT imaging, the EDL was sutured with no. 0 Fiberwire (Arthrex, Naples, FL) and fixed to the clamp of a universal testing machine (EZ graph, Shimadzu). After fixation of the tibiae using iron clamps with a rough surface, a preload of 1 N was applied. The direction of the pulling force was carefully mated with the long axis of the bone tunnel by multidirectional inspection. The bone and tendon complex was loaded at a rate of 1 mm/sec until the tendon ruptured or was pulled out. Maximum pullout force was recorded. For the pullout strength assessment, we excluded tendon rupture cases because the true interface strength could not be measured. The pullout strength of the pullout cases was compared between the two groups. The pullout strength was calculated by dividing the maximum pullout force by the interface contact area using the following formula: pullout strength (N/mm^2^) = maximum pullout force (N)/interface contact area (mm^2^). The interface contact area was calculated by multiplying the tunnel length by the perimeter of the tendon, which was assumed constant throughout the tunnel. All image analysis and biomechanical tensile tests were performed by two engineering specialists, who were not coauthors.

### 2.9. Statistical Analysis

SPSS Ver.17.0 (IBM, Chicago, IL) was used for statistical analysis. For ALP activity measurement, differences between the groups were tested with Mann-Whitney *U* tests. For image analysis and biomechanical tensile tests, differences between the groups were tested with Wilcoxon signed-rank tests. *P* < 0.05 was considered as statistically significant.

## 3. Results

### 3.1. Osteogenic Potential of the Osteogenic Matrix Cell Sheets

**Figure 2:**
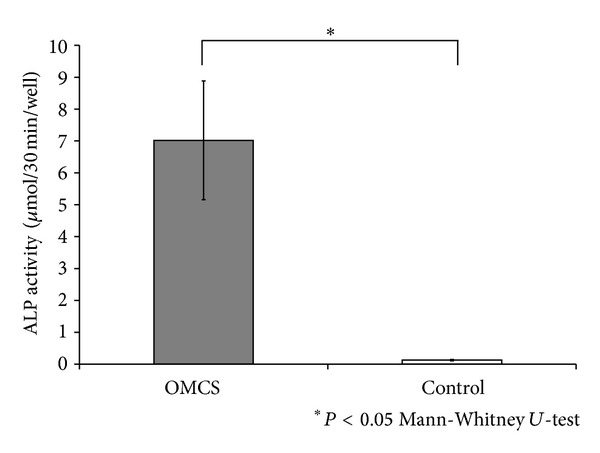
ALP activity measurement. The OMCS group showed significantly higher ALP activity than the control group (*n* = 4). Values are shown as mean ± standard deviation. Asterisk indicates *P* < 0.05.

**Figure 3:**
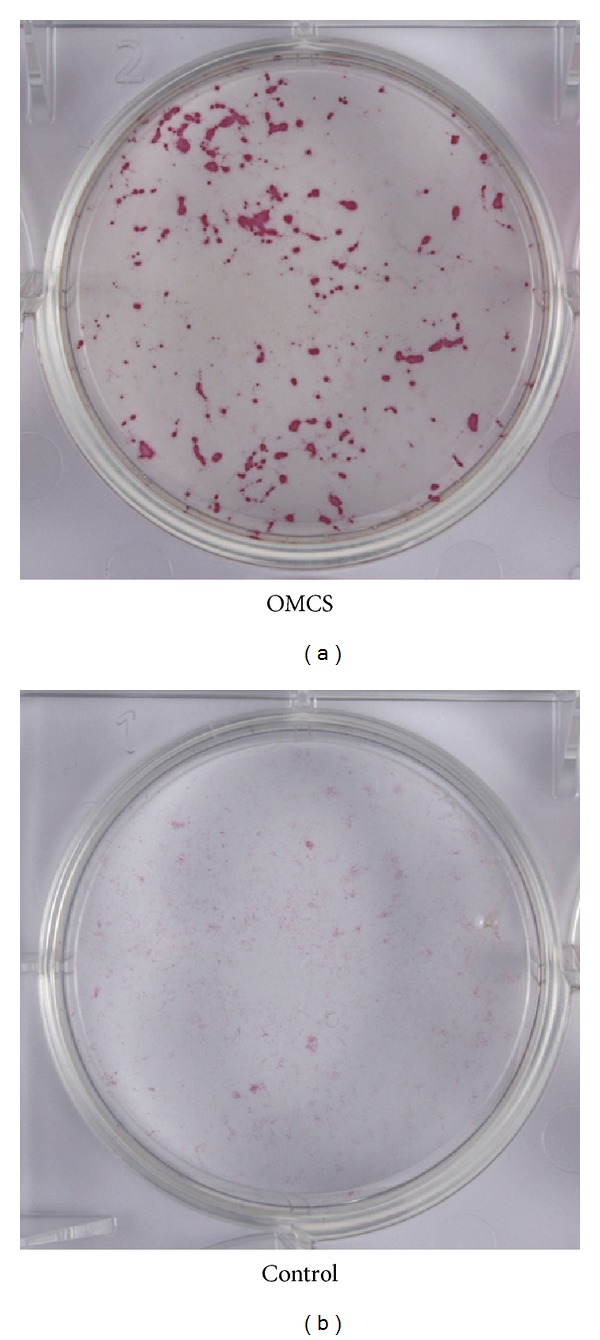
ALP staining. The ALP staining was positive in the OMCS group (a), whereas it was weak in the control group (b).

**Figure 4:**
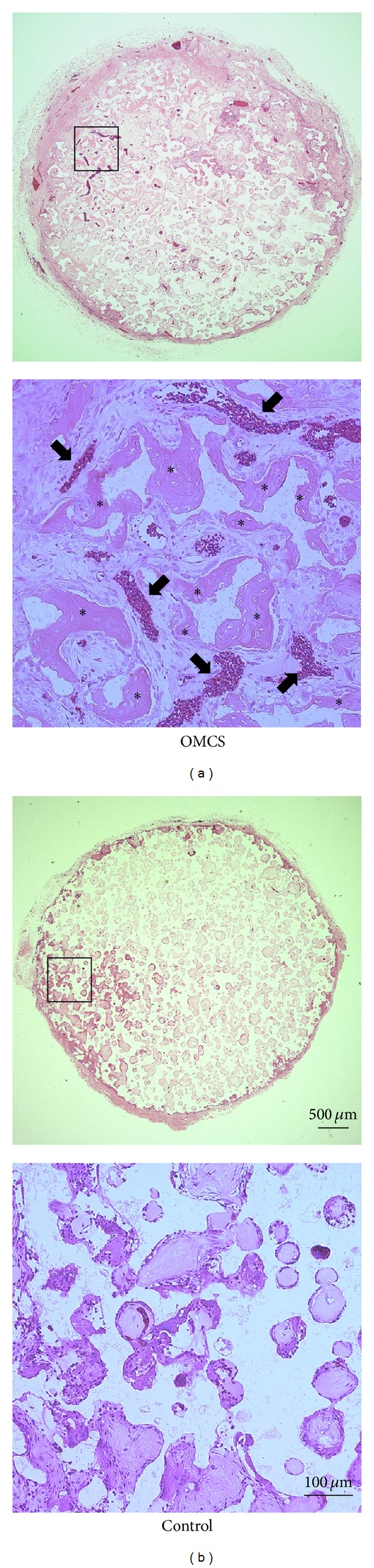
Histology of the harvested *β*-TCP constructs. Abundant new bone formation with angiogenesis was observed in the *β*-TCP construct with OMCS (a), whereas there was no bone formation in the control group (b). Asterisks and arrows indicate bone tissue and vessels, respectively.

 The OMCS showed significantly higher ALP activity than control in vitro (Figure 2). ALP staining was strongly positive in the OMCS group, whereas it was very weak in the control group (Figure 3). Histology showed that abundant new bone formation with angiogenesis was observed for *β*-TCP construct with OMCS, whereas there was no bone formation in the control group (Figure 4). The volume of new bone in the OMCS group was 38.2 ± 5.9% (average ± S.D.).

### 3.2. Histology and Immunohistochemistry of Tendon Graft to Bone Tunnel Healing

**Figure 5:**
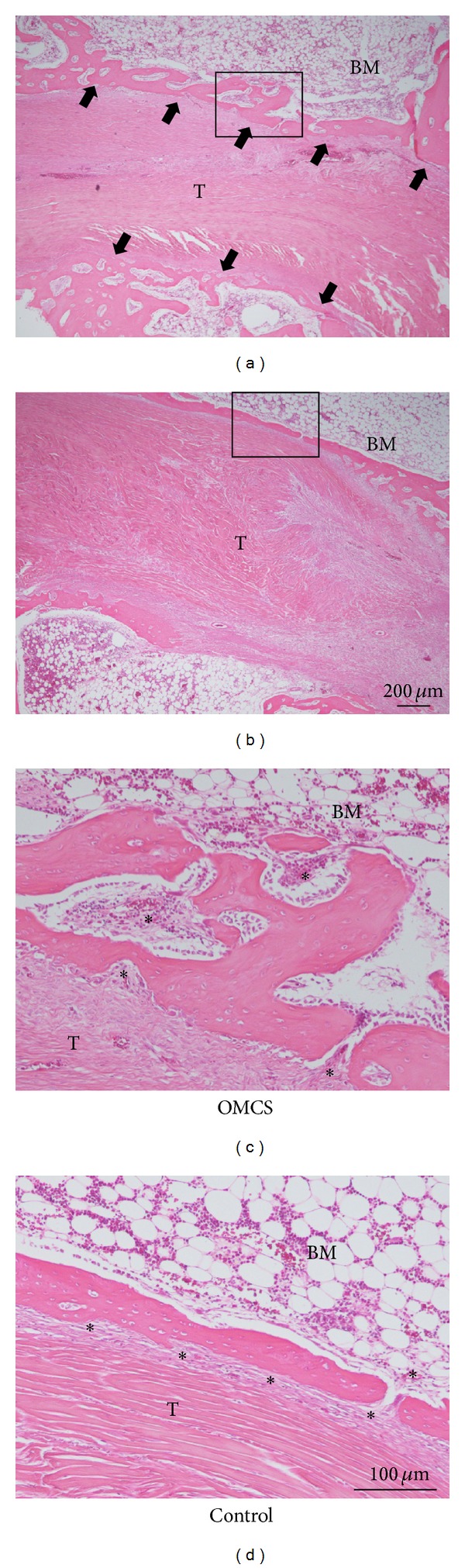
Histology of the tendon to bone tunnel interface. Thick bony walls (arrows) were observed around the grafted tendon in the OMCS group 3 weeks after the operation ((a), (c)), whereas such bony walls were thinner or seen less frequently in the control group ((b), (d)). In both groups, fibrovascular tissues were observed. (c) and (d) are magnifications of (a) and (b). T and BM indicate grafted tendon and bone marrow, respectively. Asterisks indicate fibrovascular tissues.

 The OMCS group showed thick bony walls between the grafted tendon and bone marrow. Portions of the interface between the grafted tendon and the bony wall were fibrovascular tissue. In other regions, the tendon directly contacted the bony wall. The bony wall adjoined the fibrovascular tissue and bone marrow on the opposite side of the grafted tendon. In contrast, bony walls in the control group were less frequent or thinner than in the OMCS group (Figure 5).

**Figure 6:**
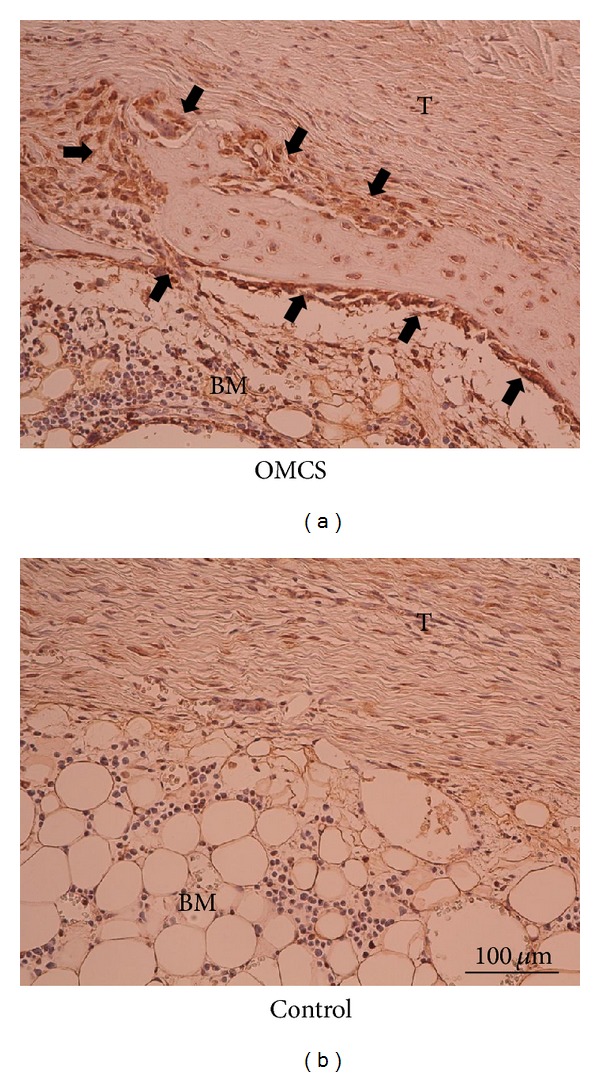
Immunohistochemistry of the tendon to bone tunnel interface for collagen type I. Positive staining was observed around the newly formed bone in the OMCS group 3 weeks after the operation (arrows in (a)). On the contrary, such strong staining was not seen in the control group (b). T and BM indicate grafted tendon and bone marrow, respectively.

Positive staining for collagen type I was detected around the newly formed bone in the OMCS group at 3 weeks after the operation. In contrast, such strong staining was not seen in the control group (Figure 6).

### 3.3. Image Analysis and Biomechanical Tensile Testing

Micro-CT image analysis showed significantly larger areas of newly formed bone around the grafted tendons in the OMCS group (4.93 ± 3.10 mm^2^) compared to the control group (3.85 ± 2.18 mm^2^) (*P* < 0.05). The major/minor axes of the cross-section of the grafted tendon were not statistically different in the OMCS group (3.61 ± 0.28 mm/2.52 ± 0.25 mm) compared with the control group (3.59 ± 0.33 mm/2.51 ± 0.27 mm). Similarly, there were no significant differences in the calculated cross-sectional area and the perimeter of the grafted tendon between the OMCS groups (7.13 ± 1.03 mm^2^, 9.60 ± 0.71 mm) and the control group (7.14 ± 0.86 mm^2^, 9.63 ± 0.56 mm), indicating equivalent grafted tendon size in both groups.

**Figure 7:**
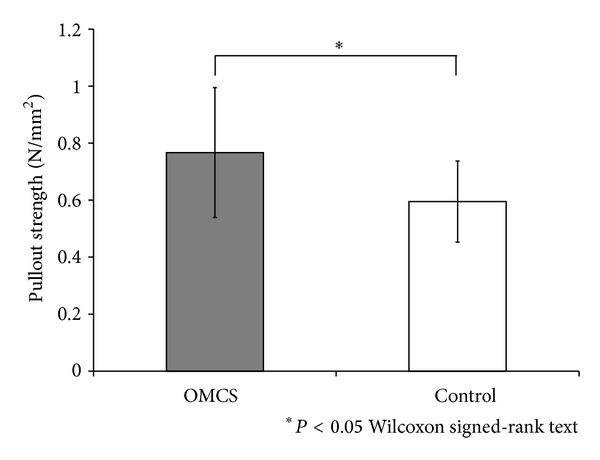
Results of biomechanical tensile testing. The OMCS group showed significantly higher pullout strength than the control group (*n* = 6). Values are shown as mean ± standard deviation. Asterisk indicates *P* < 0.05.

Of the 10 pairs of harvested specimens, the pullout strength could not be measured in four pairs owing to specimen rupture at the tendon itself (one in the OMCS and four in the control group). Therefore, six pairs of harvested specimens were used for calculation of pullout strength in the present study. Maximum pullout forces and the tunnel lengths measured by micro-CT were 39.3 ± 13.3 N and 5.4 ± 0.7 mm, respectively, in the OMCS group, and 29.2 ± 7.6 N and 5.2 ± 0.7 mm, respectively, in the control group. Thus, the calculated pullout strengths were 0.74 ± 0.23 N/mm^2^ and 0.58 ± 0.15 N/mm^2^ in the OMCS and control group, respectively (*P* < 0.05) (Figure 7). The pullout strength of the OMCS group was increased 27.2% compared with that of the control group.

## 4. Discussion

These results demonstrate that OMCS created from rabbit MSCs have osteogenic potential in vitro and in vivo. We previously reported the osteogenic potential of rat OMCS by showing that the scaffold-free transplantation of OMCS could enhance femoral fracture union in the rat [16]. However, the osteogenic potential of rabbit OMCS had not previously been evaluated. Here, we found that surrounding the grafted tendon with OMCS before transplantation induced bone tissue formation that resulted in a mechanically stronger interface by pullout strength testing. Therefore, our results suggest that OMCS transplantation could enhance tendon to bone healing around the grafted tendon in ligament reconstruction surgery.

In a canine extra-articular tendon-bone healing model, Rodeo et al. [3] described a thin seam of new bone lining the bone tunnel at 4 weeks after the surgery. At 8 weeks, collagen fibers similar to “Sharpey's fibers” were seen spanning the interface between the grafted tendon and the bone tunnel. Those fibers were anchored in the surrounding newly formed bone. Therefore, the aim of many past approaches has been enhancement of osteogenesis around the grafted tendon to accelerate the healing process [6–12]. In the present study, OMCS likely facilitated tendon to bone tunnel healing by promoting rapid production of new bone around the grafted tendon in the early stages after the operation.

Many previous reports have described various methods to accelerate tendon to bone healing. Growth factors such as bone morphogenetic protein (BMP) have been vigorously investigated [9–11]. Rodeo et al. [10] reported significant bone formation in the tendon bone interface of the canine model using recombinant human BMP-2 (rhBMP-2) at 2 weeks, which resulted in greater pullout strength. However, absorption of bone was observed because of excessive dosage of BMP-2, indicating that controlling the dosage might be difficult. In addition, the half-life of growth factors is short; multiple administrations or special techniques such as gene transfer by viral infection would be necessary. By contrast, our technique using OMCS can achieve new bone formation without growth factors or gene transfer.

Recently, MSCs have been widely investigated with a variety of scaffolds as a cell source for tissue regeneration. In general, natural or synthetic materials are used as scaffolds for MSC transplantation. However, some scaffolds may cause immunological responses or have poor bioactivity and biocompatibility [13]. We think that the scaffold-free cell transplantation technique is ideal, particularly in ligament reconstruction surgeries, because it is often difficult to fit the MSCs/scaffold construct to the interface of tendon and bone tunnels. Our method using OMCS transplantation can be easily applied to the tendon graft model. OMCS is very soft, flexible, and easily molded, resulting in a good fit between the interface of the grafted tendon and bone tunnel, as well as between tendon and tendon in folded tendon grafts. Arthroscopic surgeries are overwhelming procedures for ligament reconstruction at present [1]. Therefore, the material properties of OMCS (very soft, flexible, and easily molded) are advantageous for transplantation through the arthroscopic portal in primary ligament reconstructions and revision surgeries, in which remaining bone stock is typically a problem and a two-step surgery is often needed.

 Chen et al. [11] reported a study on photoencapsulation of BMP-2 and periosteal progenitor cells for tendon to bone healing with a very similar operative procedure as ours. They conducted biomechanical tensile tests at 3 and 6 weeks, though all specimens at 6 weeks after the operation ruptured at the tibial insertion site or midsubstance. Therefore, in the present study, we conducted the biomechanical tensile tests at only 3 weeks after the operation. However, the test failed in four of ten specimens because of rupture. Still, the results from intact specimens showed that OMCS transplantation significantly enhanced tendon to bone healing during the early stages after the operation.

There are several limitations to this study. First, the origin of the newly formed bone around the grafted tendon was not investigated in detail. Future studies using a cell labeling technique, such as transfection of a fluorescent marker protein, are needed. To complement the collagen type I data, which shows a major organic component of osteogenesis [23] that is predominantly distributed in bone and tendon [24], immunohistochemistry of other molecules related to osteogenesis, such as BMPs, should be investigated [26]. Second, although our results showed the possibility of the sheets enhancing tendon to bone healing by forming new bone, the effects of any remodeling of the grafted tendon itself were not evaluated. Remodeling of the tendon itself after transplantation is also a major weak point in ligament reconstruction surgeries. Third, we used an extra-articular model of tendon reconstruction. For the next step, it will be necessary to use an intra-articular experimental model. Fourth, these results were only from one species at 3 weeks after surgery, a relatively short follow-up time. Further experiments, including longer terms and different species, are needed. Despite these limitations, the present study clearly indicated the possibility that OMCS transplantation would be a useful technique for tendon reconstruction surgery.

## 5. Conclusion

Our study demonstrated that OMCS transplantation formed new bone tissue around the grafted tendon, which resulted in a mechanically stronger interface by pullout strength testing, promoting early tendon graft to bone tunnel healing. Application of our method to ligament reconstruction surgery could enhance early healing of tendon to the bone tunnel and accelerate postoperative rehabilitation.
